# Pathogenetic Mechanisms Linking Sarcoidosis to Lymphoma

**DOI:** 10.3390/ijms26020594

**Published:** 2025-01-12

**Authors:** Styliani Voutidou, Dimitrios Eleftheriadis, Fotios Drakopanagiotakis, Ilias C. Papanikolaou, Paschalis Steiropoulos

**Affiliations:** 1 Department of Respiratory Medicine, Medical School, Democritus University of Thrace, University General Hospital Dragana, 68100 Alexandroupolis, Greece; stella.voutidou@gmail.com (S.V.); fdrakopanagiotakis@gmail.com (F.D.); 2 Department of Respiratory Medicine, Kerkyra General Hospital, 49100 Corfu, Greece; icpapanikolaou@hotmail.com

**Keywords:** sarcoidosis, lymphoma, differential diagnosis, immunopathogenesis, genetics, risk factors, immunosuppression

## Abstract

Sarcoidosis and lymphoma share immunopathological characteristics that suggest a complex, interconnected relationship. This article examines the multi-faceted mechanisms linking sarcoidosis to lymphoma, a phenomenon called sarcoidosis-lymphoma syndrome (SLS). SLS is hard to diagnose, requiring distinct criteria and imaging to differentiate overlapping features and histological differences. The co-occurrence of these diseases may be explained by genetic predispositions, immune dysregulation, and environmental factors that enhance malignancy risk. In active sarcoidosis, chronic inflammation and granuloma formation induce the production of cytokines that can contribute to lymphoma development. The role of macrophage polarization is also discussed. Immunosuppressive treatment prescribed in sarcoidosis patients, particularly corticosteroids and biological agents, may increase the susceptibility to lymphoproliferative malignancies. These common mechanisms emphasize the need for vigilant monitoring of lymphoma in patients with sarcoidosis, as this granulomatous disease can mimic and promote the development of lymphoma.

## 1. Introduction

Sarcoidosis-lymphoma syndrome was first described in 1986 by Brincker in a study of 46 cases that demonstrated a relationship between sarcoidosis and the development of lymphoproliferative disease. Lymphoma may develop either years after a sarcoidosis diagnosis or even before. Additionally, cases have been reported in which sarcoidosis coexists with lymphoma, presenting as a paraneoplastic syndrome [1]. The average interval between the onset of sarcoidosis and lymphoma is estimated to be 24 months, although cases have been documented where the interval spans decades. Middle-aged individuals with chronic active sarcoidosis have a five-fold higher incidence of lymphoproliferative diseases, with half of these cases involving low-grade lymphomas located in the lungs. Solid tumor frequency is also elevated, particularly involving the cervix, liver, lungs, skin, testicles, and uterus [1].

The median age of sarcoidosis onset in patients who develop lymphoma is over 40 years, approximately 10 years older than in those with sarcoidosis unaccompanied by cancer. Other hematologic malignancies documented in patients with a prior sarcoidosis diagnosis exceeding one year include myeloma, acute myelogenous leukemia, hairy cell leukemia, chronic myelogenous leukemia, Epstein-Barr virus-associated lymphoproliferative disease, lymphoproliferative disorders, myeloproliferative disorder, chronic lymphocytic leukemia, mycosis fungoides, and T-cell granular lymphocytic leukemia [2]. In this paper, we review the evidence linking the pathogenesis of sarcoidosis to lymphoma.

## 2. Epidemiology

Various hypotheses describe the complicated relationship of sarcoidosis and lymphoproliferative diseases [3,4]. Most cases of sarcoidosis after lymphoma are mild or self-healing [4].

A meta-analysis of studies conducted in Japan, the UK, the USA, and Scandinavia reported a pooled relative risk (RR) of 1.19 (95% CI, 1.07–1.32) for all invasive cancers [5]. The analysis revealed an approximately twofold increased risk of developing hematologic cancers (lymphomas, Hodgkin’s lymphoma, leukemia) [5].

In sarcoidosis-lymphoma syndrome, lymphoma typically appears 2–8 years after sarcoidosis diagnosis, primarily in chronic sarcoidosis patients [3,6]. Spleen and bone marrow involvement in sarcoidosis are considered risk factors for hematological malignancy development [3,7]. Sarcoidosis-Lymphoma Syndrome (SLS) refers to the increased risk of developing lymphoma in people with sarcoidosis, a connection first recognized in 1986 by Brincker. Research shows that sarcoidosis patients are 5.5 to 11 times more likely to develop lymphoma than the general population, with one study identifying SLS in nearly 5% of sarcoidosis cases over 13 years [8]. A recent analysis reported that 2.2% of the sarcoidosis patients developed hematologic malignancies, with 1.5% developing non-Hodgkin’s lymphoma and 0.3% developing Hodgkin’s lymphoma [9]. Diffuse large B-cell lymphoma (DLBCL), a type of non-Hodgkin’s lymphoma, was the most common type observed, although both Hodgkin’s and other types of non-Hodgkin’s lymphomas can occur [10]. Other studies have confirmed the increased incidence of non-Hodgkin’s lymphoma [3,11]. In a meta-analysis, sarcoidosis was associated with an almost three times higher risk of Hodgkin’s lymphoma and a 1.43 times increased risk of non-Hodgkin’s lymphoma [5]. A recent meta-analysis reported an overall RR of 3.67 (2.92–4.63) for lymphoma in patients with sarcoidosis [9]. In this study, non-Hodgkin’s lymphoma was reported more frequently both in the early, one-year period and in the period one year after sarcoidosis diagnosis [9].

SLS typically affects individuals diagnosed with sarcoidosis around the age of 48, with no apparent gender preference [10]. Lymphoma often develops years after the sarcoidosis diagnosis, with a median interval of 11 years, although cases with simultaneous diagnoses have been reported [9,10,11]. Increased lymphoma risk has been described even in the one-year period after sarcoidosis diagnosis [9]. Diagnosing SLS can be difficult since both conditions share symptoms such as lymphadenopathy and systemic illness, making biopsy essential. While sarcoidosis patients face an elevated risk of lymphoma, their lymphoma outcomes are similar to those without sarcoidosis [12]. The underlying link remains uncertain but may involve chronic immune system activation. These findings underscore the need for careful monitoring of sarcoidosis patients to ensure timely diagnosis and treatment of lymphoma, which is crucial to improving outcomes [10].

## 3. Differential Diagnosis

Establishing a definitive diagnosis of sarcoidosis-lymphoma syndrome through clinical and radiological findings is challenging, and an optimal diagnostic approach has yet to be determined. Sarcoidosis is notable for its wide range of clinical presentations. Whether symptomatic or asymptomatic, acute or chronic, sarcoidosis can affect various organs, with clinical impacts ranging from benign to severe [13].

The diagnosis of sarcoidosis is based on three primary criteria: a compatible clinical presentation, evidence of non-caseating granulomas on histological examination, and the exclusion of alternative diagnoses [14]. On the other hand, lymphoma diagnosis is established through a lymph node biopsy, which utilizes morphological analysis, immunohistochemistry, and flow cytometry. Although fine-needle aspiration and core needle biopsy are often employed during the initial evaluation of adenopathy, these methods do not provide sufficient tissue for a definitive lymphoma diagnosis, particularly for verifying Hodgkin’s lymphoma, which requires the identification of Reed-Sternberg cells [15].

CT imaging is the preferred method for evaluating sarcoidosis and differentiating it from other mediastinal abnormalities based on mediastinal lymph node enlargement. Previous studies have highlighted the critical role of CT in diagnosing both sarcoidosis and Hodgkin’s lymphoma by assessing mediastinal lymph node enlargement. However, these studies primarily focused on qualitative analysis of imaging characteristics, rather than quantitative assessments. Significant differences exist between Hodgkin’s lymphoma and sarcoidosis regarding mediastinal lymph node enlargement. Sarcoidosis is characterized by non-caseating granulomas composed of epithelioid cells, whereas lymphomatous nodes primarily consist of lymphocytes and Reed-Sternberg cells. Consequently, distinct absorption characteristics can be observed in imaging, reflecting the histological differences between Hodgkin’s lymphoma and sarcoidosis [16].

Both sarcoidosis and lymphoma can lead to increased FDG uptake in mediastinal lymph nodes; thus, FDG-PET/CT cannot eliminate the need for histological verification [17]. In the case of Hodgkin Lymphoma, accurate interpretation of FDG-PET/CT findings is essential for determining the prognosis of the disease and selecting appropriate treatments. FDG-PET/CT scans post-chemotherapy showed persistent symmetrical bilateral mediastinal and hilar lymphadenopathy, coupled with interval progression in metabolic and morphologic features, as well as associated lung parenchymal changes, further supporting the diagnosis of concurrent sarcoidosis [18].

Ultrasound features of lymph nodes (LNs) observed through endoscopic ultrasound (EUS), combined with fine needle aspiration (FNA), provide a less invasive alternative for diagnosing the etiology of mediastinal lymphadenopathy (MLAD). While benign MLAD tends to involve smaller lymph nodes than other etiologies, ultrasound features alone are unreliable diagnostic tools. Therefore, FNA is recommended whenever feasible. However, due to the relatively low sensitivity of FNA, lymph nodes with benign FNA results should undergo further evaluation if clinical suspicion persists [19].

Previous studies have successfully utilized MRI, particularly diffusion-weighted imaging (DWI) and apparent diffusion coefficient (ADC) measurements, to differentiate between malignant and benign lymph node pathologies. Diffusion reflects the mobility of water molecules. Despite its utility, MRI does not effectively distinguish between neoplasm subtypes. Findings confirm that ADC values in lymph nodes affected by malignant lymphoma are significantly lower than those in nodes affected by sarcoidosis. Diffusion measurements also revealed lower values in sarcoidosis-affected nodes than those with malignant lymphoma. MRI-based diffusion and T2 ratio indicators facilitate differentiation between sarcoidosis-related and lymphoma-related lymphadenopathy. While the T2 ratio demonstrated higher specificity but lower accuracy than ADC values, their combined application offers a valuable diagnostic advancement. This distinction is particularly significant for younger patients presenting with similar symptoms of sarcoidosis or lymphoma [20].

Accurate diagnosis is crucial, as lymphoma and sarcoidosis can present with B symptoms, diffuse lymphadenopathy, and hepatosplenic involvement. Differentiating between these two diseases is particularly challenging, as lymphoma and sarcoidosis can rarely coexist [21].

One of the prominent features of sarcoidosis is high angiotensin-converting enzyme (ACE) levels [3]. According to a study conducted by Cerri et al., patients with SLS exhibit even higher levels of ACE than patients with sarcoidosis or lymphoma alone [12]. This phenomenon may be explained by the hypothesis that ACE reflects the intensity of lymphocytic activation. During the follow-up period of patients with sarcoidosis, excessively high levels of ACE should raise suspicion of malignancy [12].

An associated lymphoma should be considered in all patients with suspected sarcoidosis, particularly those who fail to respond to treatment or who exhibit persistent hematological abnormalities. In cases of splenomegaly, splenectomy should be performed to rule out lymphoma if less invasive methods do not provide a definite diagnosis [22]. The persistence and progression of lymphoma following the discontinuation of prednisone may contribute to the recurrence of sarcoidosis.

## 4. Immunopathogenesis

### 4.1. Granuloma Formation in Sarcoidosis and Lymphoma

According to published literature, sarcoidosis arises in genetically predisposed individuals due to a cell-mediated immunological reaction to one or more antigens, most of which are analyzed in this article. Well-formed granulomas result from this cell-mediated response to antigenic stimuli [23].

From an immunopathogenic perspective, granuloma formation represents a pathological reaction initiated by CD4+ T cells interacting with antigen-presenting cells. When antigens are encountered, they are phagocytosed and presented by cells like macrophages or dendritic cells to CD4+ T helper cells. This interaction triggers an immune response characterized by a strong Th1-type cytokine cascade, including interleukin (IL)-2, tumor necrosis factor (TNF-α), and other contributors like T regulatory cells (Tregs), which also produce interferon-gamma (γ-INF). The release of γ-IFN and TNF-α subsequently drives macrophage accumulation, activation, and clustering, leading to granulomatous inflammation. Granulomas form as a barrier around antigenic material, with a layered structure: the core contains macrophages, epithelioid cells, and multinucleated giant cells, surrounded by CD8+ and CD4+ T cells, B cells, monocytes, mast cells, and fibroblasts. These are further encased in rings of hyaline collagen. Overall, granuloma initiation and disease progression are driven by Th1 cytokines, while dysfunctional Tregs (which support immune regulation) and enhanced Th17 response may contribute to granuloma persistence [24].

Sarcoid granulomas can develop in patients with malignant tumors, such as carcinoma and malignant lymphoma, by establishing a cell-mediated immune response against tumor antigens produced by granulomas within the cancer. Sarcoidosis and lymphoma share numerous immunological traits, including cutaneous anergy, peripheral lymphadenopathies, and increased T-helper cell infiltration in tissues [25]. Additionally, patients with chronic sarcoidosis have a higher likelihood of developing lymphoproliferative diseases, most likely due to immunological abnormalities, such as B-cell system hyperactivity, a decrease in circulating T-helper cells, and an increase in T-helper cells in granulomatous tissues [26]. Sarcoidosis is frequently associated with hypergammaglobulinemia, suggesting B-cell hyperreactivity in up to 80% of cases [27].

Different types of lymphomas are often sites of granuloma formation [28]. Among others, authors have described the involvement of classical Hodgkin lymphoma (cHL), adult T-cell leukemia/lymphoma, angioimmunoblastic T-cell lymphoma, T-cell/histiocyte-rich B-cell lymphoma (THRBCL), chronic lymphocytic leukemia/small lymphocytic lymphoma, lymphoblastic lymphoma, nodular lymphocyte predominant Hodgkin lymphoma, and peripheral T-cell lymphoma, particularly the Lennert variant [29]. HL is more frequently associated with granuloma reaction (9–29% of cases), while they were detected in up to 7.3% of patients with NHL [28].

Although the exact mechanisms involved are not yet fully understood, prolonged lymphoma cell antigenemia, macrophage activity, Th1 cell response, and different biological mediators have been implicated. Neoplastic antigens can trigger a T-helper-mediated hypersensitivity reaction, activating monocytes and forming epithelioid histiocytes [28]. These histiocytes, which adopt an epithelioid morphology, manifest as well-formed granulomas [29].

Cytokines produced by tumor cells (i.e., in Hodgkin or T-cell lymphoma) or cells in their microenvironment can evoke granulomatous inflammation. Type 1 cytokines, such as interferon-gamma, are mainly involved in granulomatosis as potent activators of macrophages and mediators in their transformation into epithelioid histiocytes and multinucleated giant cells [28].

Sometimes, the intense granulomatous reaction can overshadow the neoplastic cells, increasing the risk of misdiagnosis and false-negative results in fine-needle aspiration (FNA) samples [28,29].

### 4.2. Pathophysiology

**Figure 1:**
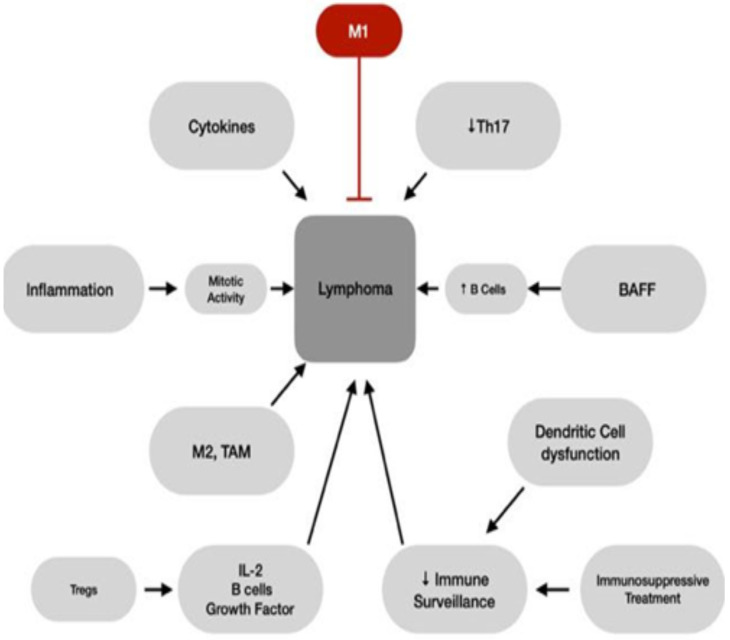
In sarcoidosis, multiple factors trigger the development of lymphoma, as depicted in this figure. The role of cytokines, TAMs, B-cell growth factors (BAFF, IL-2) is highlighted. Decreased immune surveillance and Th17 response may also increase the risk for malignancy, while M1 macrophages seem to have a protective effect. BAFF: B-cell activating factor, IL-2: interleukin 2, M1: M1 macrophages, M2: M2 macrophages, TAM: tumor-associated macrophages.

Immunopathogenic pathways in both sarcoidosis and cancer involve immune dysregulation and chronic inflammation as the primary triggers [24,25]. Some of these mechanisms are discussed below and summarized in Figure 1.

Three mechanisms have been proposed to broadly explain the relationship between sarcoidosis and cancer [24].

1. Genetic Predisposition and Environmental Triggers: Genetically predisposed individuals with sarcoidosis may develop malignancies after persistent environmental exposure;
2. Immunosuppressive Treatment Risks: Corticosteroid treatment, often required in sarcoidosis, can reduce immune surveillance, potentially increasing cancer risk (discussed further in a later chapter);
3. Sarcoid-Like Reactions (SLRs): Some malignancies can trigger sarcoidosis-like responses in tissues without systemic sarcoidosis, notably when malignancy predates sarcoidosis [24].

Inflammatory cytokines (e.g., tumor necrosis factor-α, interleukin-6, and transforming growth factor-β, nitric oxide, and vascular endothelial growth factor) can increase the risk of malignancy when produced excessively, as they may promote angiogenesis, cellular proliferation, stromal growth, and tissue remodeling. Dysfunctional myeloid dendritic cells may further impair tumor immune surveillance [30,31,32].

The role of macrophage polarization is also significant. The macrophage is a common dominator in inflammation and tumor formation processes and engages in innate and adaptive immune response [33]. The states of homeostasis, chronic inflammation, and disequilibrium are regulated by the dynamic switch between M1 and M2 polarization [24].

M1 (“killer”) macrophages, the first line of defense against intracellular pathogens, induce Th1 CD4+ response and complement-mediated phagocytosis. The suitable level of CD64 and CD80 markers is expressed according to the stimuli. Various antimicrobial mechanisms are also activated due to the production of proinflammatory agents, e.g., cytokines, chemokines, reactive oxygen, and nitrogen intermediates. Eventually, the M1-like macrophage eliminates the antigen, resolves the inflammation, and induces cancer cell cytolysis [34].

M2 (“healer”) macrophages (CD163+) produce anti-inflammatory cytokines, such as IL-4, IL-10, and TGF-β, to modulate the inflammation and protect the organism [35]. Repair mechanisms, metabolic processes, and granulomatosis formation are other pathways these macrophages contribute to [24]. In advanced stages of sarcoidosis, M2 activation can even cause fibrosis [36,37].

The subpopulations of these cells are of particular interest in the topic of malignancies, even though their role usually involves parasitic, helminthic, and fungal infections [35]. The M2d population, called Tumor-Associated Macrophages (TAMs), arises from adult myeloid precursors in circulation and may induce an inflammatory response or its resolution. The different behaviors of TAMs depend on reprogramming, continuous plasticity, and the present stimuli that conclude with their self-regulating polarization. The reprogramming of an immunosuppressive microenvironment results in cancer cell proliferation, invasion, and metastasis [38].

The specific equilibrium between M1 and M2 polarization is unique for different neoplasms and is decisive for tumor progression [39,40].

M1- and M2-macrophages have distinct roles in the sarcoidosis microenvironment. M1-polarization’s anticancer properties in SLR may be a natural barrier to a neoplasm. M2-like populations, on the other hand, can lead to hematological lymphoproliferation, such as lymphoma, MGUS, and macroglobulinemia [24]. For lymphoma formation in particular, activating the lymphocyte-macrophage axis—such as the one observed in active sarcoidosis—is a primary driver of malignant proliferation [4,10].

Elevated mitotic activity and unregulated cellular proliferation can occur after a specific trigger [24], increasing simultaneously the risk for mutation and malignant transformation [2]. In sarcoidosis, lymphocytes undergo multiple mitoses in response to inflammation [2]. In addition, BAFF, a pro-proliferative cytokine for B lymphocytes, is elevated (in correlation with ACE levels) and could trigger clonal proliferation [6,24]. Interestingly, in patients with sarcoidosis-lymphoma syndrome, BALF has a higher CD4/CD8 lymphocyte ratio than in other patients [6].

According to another hypothesis, sarcoidosis induces regulatory T-cell amplification. As a result, naive and effector T cells proliferate and increase the production of IL-2, a B cell growth factor. This might explain the observation of altered B cell subtypes in the peripheral blood of sarcoidosis patients [41].

In a study conducted by Hachisu et al. (2022), the potential role of Th17-dominant immune response was discussed. Th17 balance seems to be elevated in tumor immunity in patients undergoing ICI treatment [42] and in BALF from active sarcoidosis [43,44]. When sarcoidosis subsides and Th17 balance lowers, a greater likelihood of neoplasm onset is observed, implying that the remission stage of sarcoidosis may be the most susceptible to tumor development.

Regarding the microenvironment of classical Hodgkin lymphoma (cHL), it is known that its composition is not limited to Reed–Sternberg (RS) and Hodgkin (H) cells, which represent just 1–10% of the total tumor mass [45]. The tumor microenvironment (TME) results from the immediate effects of cancer cells on their surroundings. It is primarily composed of non-malignant T and B lymphocytes, plasma cells, histiocytes/macrophages, granulocytes, eosinophils, mast cells, mesenchymal stromal cells, and endothelial cells [28,45,46]. The proposed mechanism of granulomatous response in cHL again includes a Th1 cell-mediated immune response to tumor antigens and hypersensitivity reactions to degenerative changes within the tumor microenvironment. Prolonged stimulation by tumor antigens can lead to fibrosis and granuloma formation [29]. The specific equilibrium between M1 and M2 macrophage polarization in the microenvironment of each neoplasm is critical for tumor progression [39,40]. Abundant macrophage infiltration, mainly tumor-associated macrophages (TAMs), often correlates with a poor prognosis [47].

Tumor-associated macrophages (TAMs) in cHL are known to express programmed cell death ligand 1 (PD-L1) likely through the activation of the JAK/STAT3 pathway [28], while RS cells overexpress PD-L1 and PD-L2 [28,45]. The immune surveillance is significantly surpassed in association with granulomas that also produce PD-L1 [28]. Consequently, immune checkpoint inhibitor treatment has been suggested to target neoplastic cells, granulomas, and the cells of the microenvironment [28,45].

The proposal mentioned above is complicated by the fact that immune checkpoint inhibitor (ICI) treatment, which includes PD-1, PDL-1, and CTLA-4 inhibitors [], is also known to cause sarcoid-like granulomas [42] through the induced predominance of Th1 response. CTLA-4 and PC-1 inhibitors provoke IL-17 secretion by CD4+ cells and increase the Th17/T regulatory cell ratio. CTLA-4 blockade also contributes to the increase in Th1-related markers [42].

## 5. Epigenetics

MicroRNAs may be linked to various forms of lymphoma and sarcoidosis. MicroRNAs (miRNAs) are small noncoding RNAs that post-transcriptionally regulate mRNAs expressed in the human genome [48].

In sarcoidosis, microRNAs regulate the TGFβ and “wingless and integrase-1” pathways [49]. The latter pathway is active during embryogenesis, tissue homeostasis, and healing [50]. Higher levels of miR-146a and miR-150, two of the most commonly studied miRNAs, are found in sarcoidosis patients compared to controls and are associated with a more severe radiologic and functional phenotype [48]. The study also found that miR-146a may act as an inhibitor, with increased expression induced by TNF-α and IL-1β in the context of NF-κB activation, while miR-150 may be involved in the activation of CD3+CD4+ T cells via NOTCH3 [48].

miR-146a is considered a tumor suppressor in various types of cancer [51] and may be associated with the pathogenesis of lymphomas. A recent study reported that miR146a may be used as a prognostic biomarker in patients with DLBCL, both at baseline and after chemotherapy, with higher levels associated with better outcomes [51]. In a miRNA analysis of patients with DLBCL, miR-146a was associated with a 13% lower hazard ratio of resistance to single-drug chemotherapy components [52]. MiR-150 is also associated with hematologic malignancies. Low miR-150 expression was present in Burkitt lymphoma cell lines, and restoration of its expression is associated with a better prognosis [53]. Lower miR-150 expression is associated with worse survival in B-cell-associated tumors [53]. Other miRNAs have been implicated in the pathogenesis of sarcoidosis and lymphoma. However, based on the current literature, common miRNAs in patients with sarcoidosis and lymphoma have not been identified.

Differentially expressed proteins (DEPs) may contribute to sarcoidosis-lymphoma syndrome. A proteomic analysis comparing the DEPs in vitreoretinal lymphoma (VRL) with those in sarcoidosis and controls identified 1594 proteins in the vitreous humor of the samples. Out of the 1594 vitreous proteins, VRL had 1571, ocular sarcoidosis had 1425, and control patients had 1163. Of these proteins, 150 were identified only in individuals with VRL, while 1138 were common proteins present in all three groups [54].

In VRL, 16 DEPs were significantly upregulated, and one significantly downregulated compared to ocular sarcoidosis [54]. In the study by Komatsu et al., the primary focus was on identifying proteomic differences between VRL, ocular sarcoidosis, and normal controls. However, some proteins were found in ocular sarcoidosis and lymphoma cases when compared to normal controls. These shared proteins indicate overlapping pathways or immune-related mechanisms between the two conditions. This overlap may suggest a common underlying immune or inflammatory response in the vitreous humor in these conditions, though the study emphasized distinct proteomic signatures for VRL and sarcoidosis [54]. Proteins like HMGB2 (related to mitochondrial energy metabolism) and PSAT1 (linked to serine biosynthesis) showed notably higher expression in VRL, suggesting a connection to tumor cell metabolism. Pathway analysis using Reactome and KEGG identified significant modifications, with the “proteasome” pathway and the “citrate cycle (TCA cycle)” among the most altered. The Reactome analysis highlighted “nuclear events mediated by NFE2L2” and changes in cell proliferation pathways, such as “programmed cell death” and “cellular responses to stimuli,” which may contribute to tumor cell growth. Key proteins involved in these changes include PSMA7, PSMB6, PSMA5, PSMA6, PSMB4, PSMA3, PSMB5, PSMA4, PSMB2, and PSMA2 [54].

Over the past two decades, studies have increasingly demonstrated that persistent oxidative stress can lead to chronic inflammation, which may underlie numerous chronic diseases, including diabetes, cancer, heart disease, neurological disorders, lung conditions, and hematologic disorders like lymphoma. Oxidative stress activates transcription factors such as NF-κB, AP-1, p53, HIF-1α, PPAR-γ, β-catenin/Wnt, and Nrf2, leading to the expression of over 500 genes related to growth factors, inflammatory cytokines, chemokines, cell cycle regulation, and anti-inflammatory responses [55].

Even though oxidative stress is not directly considered a genetic factor, genetics can significantly influence the cell’s susceptibility to oxidation. In sarcoidosis, oxidative stress plays a key role in pathophysiology and disease progression. Elevated oxidative stress markers, such as oxidatively damaged macromolecules and lipid peroxidation products, have been reported in sarcoidosis patients, generating interest in non-invasive predictive tools for disease outcomes. Disruptions in antioxidant defense systems, such as glutathione, superoxide dismutase, and paraoxonase-1, are linked to disease development. Disturbed mitochondrial homeostasis, leading to reactive species, also impacts cellular health [56]. Oxidative stress plays an important role in lymphoma pathogenesis. Tumor cells tend to produce energy in the initial stages through glycolysis rather than aerobic cellular respiration, even in the presence of oxygen. This leads to an oxidative cell environment [57]. Mitochondrial damage and excessive superoxide production occurs, with an effect of oxidative stress on DNA, cell proteins, and lipids, which are important steps to carcinogenesis [57]. Through the activation of multiple transcription factors, oxidative stress can produce a chronic inflammatory state by boosting the production of proinflammatory cytokines, excessively stimulating B lymphocytes to produce antibodies, and potentially altering cellular DNA [57]. Hypothetically, the increased oxidative stress seen in sarcoidosis might affect, when out-of-proportion cell metabolism and predispose to carcinogenesis. Since lymph nodes are the most common site of sarcoidosis involvement, oxidative stress might preferentially affect lymphocytes, thus connecting sarcoidosis and lymphoma. Epigenetic mechanisms, including DNA and histone methylation and molecular pathways like JAK/STAT, may further contribute to sarcoidosis development, although additional research is needed [58,59]. Epigenetic mechanisms, including DNA and histone methylation, are also implicated in lymphoma. Moreover, abnormal JAK/STAT signaling is observed in various T-cell malignancies, suggesting another potential link to sarcoidosis [60,61].

### Genetics

Despite the epidemiological evidence linking sarcoidosis to lymphoma, a recent mendelian randomization study examining the effect of autoimmune diseases on the risk of non-Hodgkin’s lymphoma came to the surprising conclusion that genetic susceptibility to sarcoidosis might protect from non-Hodgkin’s lymphoma. However, this association was no longer significant after accounting for multiple tests [11]. Such studies highlight, however, the uncertainty about the presumed genetic linkage of the two diseases. Other studies have reported that the MyD88 gene single nucleotide polymorphisms −938C>A (dbSNP rs4988453) and 1944C>G (dbSNP rs4988457) have been associated with sarcoidosis development [62]. These haplotypes have been associated with the development of Hodgkin’s lymphoma [63]. has been associated with sarcoidosis, with different alleles linked to distinct sarcoidosis phenotypes, such as DRB103 with Löfgren’s syndrome and DRB1*04 with ocular sarcoidosis [64,65]. A linkage to DRB1 is a shared feature of sarcoidosis and lymphoma. In some cases, the allele status is similar between sarcoidosis and lymphoma: the HLA-DRB1*07 is associated with an increased risk for sarcoidosis and Hodgkin’s lymphoma [66,67]. This also applies to HLA-DRB1*11 and HLA-DRB1*12 and Epstein-Barr virus-associated lymphoma [67,68].

**Figure 2:**
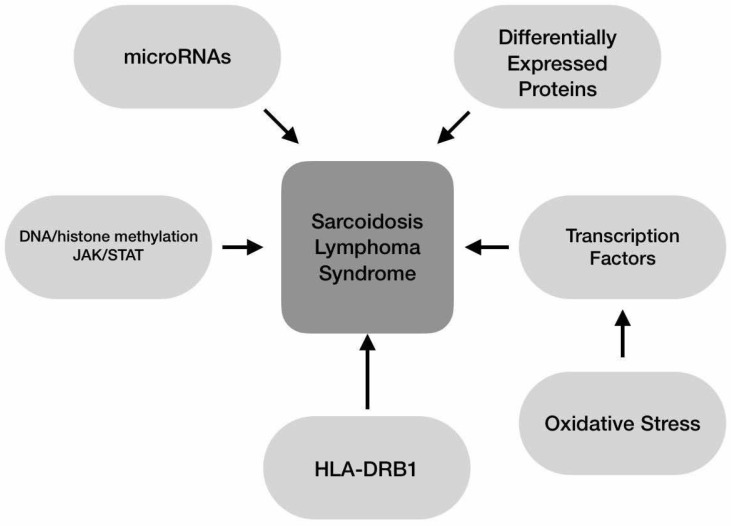
Genetic factors significantly influence sarcoidosis-lymphoma syndrome, with molecular interactions shaping immune responses. MicroRNAs regulate gene expression, while DNA and histone methylation affect chromatin structure and gene activity. The JAK/STAT pathway drives cytokine-mediated inflammation, and HLA-DRB1 variations highlight its role in antigen presentation. Protein expression and oxidative stress via transcription factors further contribute to immune dysregulation and tissue damage, advancing disease progression.

Mechanisms that may connect sarcoidosis to lymphoma are summarized in Figure 2.

## 6. Environmental and Infectious Factors

### 6.1. Common Environmental Triggers

Several studies have reviewed the environmental factors that predispose individuals to or prevent the formation of lymphoma and sarcoidosis separately.

Four different pathophysiological pathways have been described by which the environment can affect the pathogenesis of sarcoidosis in particular [69].

The first mechanism involves exposure of the organs—mainly the lungs and skin—to the antigen, which is captured and bound to the HLA II molecules of APCs. The antigen is then presented to CD4+ T-cells, resulting in a Th1/Th17 response and, ultimately, the formation of sarcoid granulomas. Various HLA polymorphisms alter this interaction and influence the disease phenotype. Specific polymorphisms can even protect the individual from the granulomatous disease [69].

The second mechanism proposes that autoreactive B- and T-cells are produced through molecular mimicry following exposure to the antigen and subsequent immune system dysregulation. Specifically, for Lofgren Syndrome (a manifestation of sarcoidosis), vimentin is believed to be the autoantigen. Overlap with certain connective tissue diseases is possible, as antinuclear antibodies have been detected in some cases [69].

The third mechanism that may explain the pathogenesis of drug-induced sarcoidosis (DISR) can be divided into two phases. In the first phase, an external factor affects the individual’s immune system, so in the second phase, it is more susceptible to another agent leading to sarcoidosis formation. For instance, in DISR, certain drugs (e.g., immune checkpoint inhibitors [ICIs]) increase the risk of sarcoidosis in “susceptible” individuals [69].

Lastly, the authors suggest that external factors can be associated with sarcoidosis without necessarily actually causing it [69]. These mechanisms are mainly proposed based on in vitro studies, and they may all contribute to persistent granulomatous disease.

In Table 1 and Table 2, the different risk and protective factors are listed.

Here, we will briefly review some of the factors that are in common between the different entities.

Firstly, the striking difference regarding the effect of smoking is worth noting. Cigarette smoking is linked to lymphoma formation (NHL and HL) and especially to the follicular form [71,72]. Not the same can be said for sarcoidosis, as it was more common for non-smokers to develop the disease [74].

Sun exposure appears to protect against both lymphoma and sarcoidosis [69,72]. Decreased sun exposure causing 1,25-hydroxyvitamin D deficiency (the biologically active form of vitamin D) has been interlinked to reduced production of cathelicidin, an antimicrobial peptide. This mechanism predisposes to infectious granulomatous diseases (e.g., tuberculosis) [69].

Moreover, those who work in the field of agriculture or live on a farm are at high risk of sarcoidosis and lymphoma. This may be due to environmental factors, such as pesticides, or infections associated with livestock farming [71]. Additionally, obesity and high BMI have a positive association with both sarcoidosis and lymphoma [71,72,73].

### 6.2. Infectious Diseases

Infectious risk factors have been associated with both sarcoidosis and lymphoma. These factors are briefly analyzed.

#### 6.2.1. NHL

Research suggests that the etiology of non-Hodgkin lymphoma involves infections—mainly viral—and immunosuppression. Human herpes virus 8, hepatitis C (HCV) [71,72], hepatitis B (HBV), human T-cell lymphotropic virus 1 (HTLV-1) [71], Epstein-Barr virus, human immunodeficiency virus (HIV), *Helicobacter pylori* (linked to gastric MALT lymphoma) [71,72] are all linked to the pathogenesis of NHL. Additionally, some bacterial agents have been suspected, such as *Campylobacter jejunii*, *Chlamydia psittaci*, and lastly, *Borrelia burgdorferi*, which is associated with rare MALT lymphomas [71].

#### 6.2.2. HL

The development of HL seems to be the outcome of the interaction of genetics, environmental factors, and the likely impaired immune system. An abnormal response of the latter to various infectious agents may trigger oncogenic processes. Although exposure to infections from a young age can be a protective factor against HL [72,73]—because of the premature maturation of cellular immunity [72] and the subsequent cytokine balance [73]—early exposure to EBV does not follow the same path. Infectious mononucleosis (IM), increases the risk for HL, and the young age of infection elevates even more the relative risk [72].

Immunosuppression also plays a role in the pathogenesis, but to a smaller extent than in NHL. HIV infection does increase the risk for HL, while the majority of the patients are also EBV positive [72]. The rise of antiretroviral therapy has decreased the number of cases caused by the virus [73].

#### 6.2.3. Sarcoidosis

Although evidence is conflicting, mycobacteria have been linked to sarcoidosis [69,75,76], with the suggested mechanism being that mycobacterial antigens—in the absence of viable mycobacterial organisms—may trigger the immune process of sarcoidosis [69]. This claim is supported by identifying different mycobacterial components [69,75]. Immune responses to mycobacteria have also been reported in BAL samples [75,76].

Bacteria (e.g., Borrelia [69], Propionibacterium acnes [69,75,76]) and fungi [69,75] are the other organisms that have been associated with sarcoidosis. Propionibacterium acnes, a skin commensal bacterium, is the only documented micro-organism cultured from sarcoid tissues. Specific immune responses to the bacterium in sarcoidosis patients have also been recognized [69,76].

Some studies suggest that alterations in gut and respiratory microbiota might play a role in the development of the disease since human microbiota contributes to immune homeostasis. A less diverse and abundant microbiota was described in sarcoidosis patients’ bronchoalveolar lavage (BAL) [69]. Other studies have also observed increased *Atopobium* and *Fusobacterium* presence in pathological BAL fluid [69,77]. Opposite results have been reported, where no significant difference between the lung microbiota of healthy and non-healthy subjects was observed [75].

While infectious agents play a role in the pathogenesis of lymphoma and sarcoidosis, a common link is not evident. Lymphoma development is more associated with viral agents and immunosuppression, while sarcoidosis seems to have a stronger link to bacterial and mycobacterial agents. *Propionibacterium* acnes antibodies have been rarely reported in patients with sarcoidosis and lymphoma, specifically MALT lymphoma [78], while sporadic reports have correlated tuberculosis infection to lymphoma [79,80]. Identification of mycobacterial genetic material in patients with lymphomas is considered an infection due to the lymphoma or the immunosuppressive therapy and is treated accordingly.

Weak evidence supports that *Borrelia burgdorferi* may cause rare subtypes of MALT lymphoma [71]. Lyme borreliosis, a multi-systemic disorder caused by Borrelia burgdorferi (Bb), has been related to NHL subtypes—and not overall risk for NHL—via self-reported history of infection and seropositivity for anti-Borrelia antibodies. Mantle cell lymphoma, a rare B-cell NHL, was the specific subtype where the link was determined [81].

In regions where Lyme borreliosis is endemic, anti-Borrelia antibodies were detected at a higher rate in sarcoidosis patients compared to healthy subjects and individuals in non-endemic areas [82]. Pathophysiological causation is considered possible by Ishihara et al., but cross-reactivity between Borrelia and other agents suspected to trigger the development of sarcoidosis (molecular mimicry) was not excluded [82]. In another study, DNA of Bb was identified using PCR in a minority of sarcoidosis patients, and a possible etiologic role was suggested for these cases [83].

EBV may be a potential link between lymphoma and sarcoid-like lesions. It was reported that sarcoidosis-like reactions favor Hodgkin lymphoma, which is often EBV-positive, suggesting that the virus may be one of the causative factors [28].

A further evaluation of infectious factors that contribute to the formation of lymphoma and sarcoidosis separately, as well as the examination of possible shared risk factors, is needed.

## 7. Effects of Immunosuppressive Treatment

Immunosuppression’s role in malignancy development, especially in organ transplant recipients, is well documented. In patients undergoing long-term immunosuppressive treatment, such as those with organ transplants, viral and non-viral agents elevate the risk of virus-related cancers and B-cell non-Hodgkin lymphoma. Newly formed neoplastic cells can no longer be detected by the patient’s immune system. In the case of transplant recipients, the risk of malignancy depends on the intensity and duration of immunosuppressive treatment [84]. Within the first year following solid organ transplantation, Epstein-Barr virus (EBV)-related diffuse large B-cell lymphoma (DLBCL) is the most common type of lymphoma associated with immunosuppression [85]. Thus, the question arises whether the immunosuppressive treatment of sarcoidosis can potentially lead to lymphoma.

### 7.1. Immunosuppressants Used in Sarcoidosis

Not all patients with sarcoidosis require treatment, as 30% of cases will subside without intervention [86]. Reducing morbidity and mortality risk and improving quality of life (QoL) constitute the treatment’s primary goals; their assessment is crucial in decision-making [87]. The guidelines also differ according to the phase of the disease (acute, chronic, or acute on chronic) [86] and its manifestations (pulmonary, cardiac, and neurosarcoidosis) [86,87].

The main treatments prescribed include:

- Corticosteroids (e.g., Prednisone): These remain the first line of treatment in patients with sarcoidosis [86,87]. The lowest possible dosage of corticosteroids (CS) is preferred for long-term maintenance, achieved by adding second- and third-line agents [86].
- Methotrexate (MTX) and anti-TNF-α antibodies (Infliximab-IFX): The second- and third-line treatments. These are used in cases of unacceptable toxicity of CS, in refractory or relapsing disease, and as steroid-sparing agents [86].
- Other drugs: Azathioprine (AZA), Leflunomide, and Rituximab, an anti-CD20 B-lymphocyte antibody used as a third-line drug [86].
- Pulmonary Fibrosis: Antifibrotic treatment (nintedanib) may be added to the therapeutic regimen, and in severe cases, lung transplantation might be necessary [86].

### 7.2. CS and Lymphoma

Although oral corticosteroids are frequently used in sarcoidosis, their role in lymphoma formation remains controversial.

Some authors have concluded that topical steroids may serve as an independent risk factor for HL that depends on the dosage, duration, and potency of the treatment [88,89]. Others have refuted this claim [85,90], while the former study accepts the association of topical treatment only due to systemic immunosuppression. These findings are worth noting in this analysis since topical therapy may be used in cases of anterior uveitis and skin lesions [91].

Regarding oral administration, there have been results in favor of their involvement in lymphoma formation [71] but also against it [84,92].

In a case-control study that collected data from the UK Clinical Practice Research Datalink (CPRD), the authors suggested that all routes of administration were associated with increased risk of lymphoma, especially HL, and in patients younger than 50 years old. Intravenous and intramuscular administration predominantly affected the relative risk, followed by oral, topical, and inhaled steroids [93].

### 7.3. MTX, Anti-TNF-α Agents and Lymphoma

The effects of MTX and anti-TNF-α were studied in patients who have rheumatoid arthritis, and no elevation of relative risk in lymphoma formation was documented [94,95,96].

Likewise, patients with inflammatory bowel disease (IBD) receiving anti-TNF-α treatment were not more at risk of lymphoma than other IBD patients [97].

Despite these observations, hepatosplenic T-cell lymphoma (HSTCL), a rare and lethal disease, was associated with IFX and primarily with thiopurine use (AZA or 6-6-mercaptopurine) [97].

### 7.4. AZA and Lymphoma

The risk of NHL and squamous cell skin cancer was found to be elevated in non-transplant patients under AZA treatment.

Azathioprine, a 6-mercaptopurine derivative, may promote cancer development via two mechanisms:

- Its immunosuppressant abilities, particularly after viral exposure in post-transplant patients, can promote lymphoproliferative disorders;
- It can directly damage DNA through 6-thioguanine accumulation [84].

Moreover, as mentioned above, AZA is also linked to HSTCL [97].

Given these variable risks associated with immunosuppressive therapies, ongoing assessment of lymphoma risk is crucial in sarcoidosis patients requiring long-term immunosuppression.

However, the reverse phenomenon has also been described. Systemic sarcoidosis or only local reactions, as described below, may occur after immuno-suppressants or chemotherapy, as published in a series of sarcoidosis flaring after breast cancer treatment [98].

## 8. Sarcoid-Like Reactions

The development of non-caseating epithelioid cell granulomas in oncologic patients not fulfilling the systemic sarcoidosis criteria is a sarcoid-like reaction [2].

Recent evidence suggests that oncological immunostimulant therapies (such as interferon, PDL1 inhibitors, and anti-TNF-α) may be responsible for granuloma formation [4,24,42], especially in patients with hematologic malignancies, such as non-Hodgkin’s lymphoma, chronic myelogenous leukemia, multiple myeloma, essential thrombocytosis, and predominantly Hodgkin’s disease [2]. When drug-induced, these reactions are known as drug-induced sarcoidosis (DISR) [24]. Alpha interferon is the most common therapeutic agent that causes sarcoidosis in oncologic patients but poses a risk factor for patients without malignancies [2,4,28].

**Figure 3:**
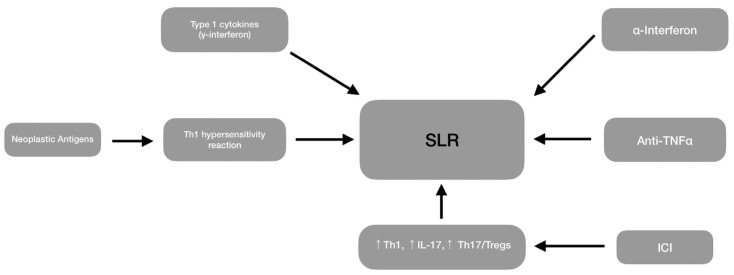
Sarcoid-like reactions can be the result of various agents, as shown in this figure. Notably, different oncological therapies, such as ICIs, anti-TNF-α, and especially α-interferon can contribute to granuloma formation. Regarding the lymphoma microenvironment, the Th1-mediated hypersensitivity reaction and cytokines (e.g., interferon-gamma) can be involved in granulomatosis. ICI: immune checkpoint inhibitors, SLR: sarcoid-like reactions, TNF-a: tumor necrosis factor a.

Figure 3 depicts the mechanisms contributing to SLRs.

This reaction should not be considered an adverse prognostic factor; conversely, it can mark a strong immune response and be a barrier to cancer cells [4,6,24]. SLR can mainly be seen in lymphoma and testicular cancer [6].

Differentiating SLR from sarcoidosis is challenging since both have been described in malignancies and are histologically indistinguishable. Their resemblance suggests a shared pathogenesis, though their initial trigger for their formation differs [99,100].

In cases of DISR, the diagnosis is confirmed by the resolution of lesions after discontinuing the treatment. In skin lesions, however, the two entities are clinically and histologically indistinguishable, and some authors suggest that these cases are considered sarcoidosis limited to the skin [101].

This differentiation is crucial for clarification of the final diagnosis and subsequently enabling more appropriate therapy, thereby improving the quality of life for patients [99].

SLRs are the perfect example of the heterogeneous nature of sarcoidosis [57,100]. Although sarcoidosis is treated as a homogeneous disease, the clinical aspect of the pathological finding of granulomas is extremely diverse [100]. It may be possible that SLRs, due to various stimuli, such as drugs, or due to microenvironment changes, as seen in malignancies, present as the most ‘limited’ form of the sarcoidosis spectrum [100]. It may be assumed that SLRs have a known association, and limited clinical sequelae and sarcoidosis, affecting pathologically and functionally several organs, present the more generalized form of this heterogeneous spectrum, while the exact antigen causing the granuloma formation remains largely unknown [100]. Big data analysis shows promising results in characterizing phenotypes and endotypes of sarcoidosis [57].

## 9. Prognosis

When diagnosed, sarcoidosis-lymphoma syndrome does not seem to alter the prognosis compared to patients with lymphoma alone. In a descriptive cohort study, a history of sarcoidosis did not seem to affect the prognosis of patients with DLBCL [10]. An Italian study of ten patients with sarcoidosis-lymphoma syndrome and 199 patients with sarcoidosis alone failed to show differences regarding survival [12]. Other studies, however, have reported a dismal prognosis in patients with sarcoidosis who develop lymphoma: in a study of 79 patients with sarcoidosis and lymphoma, fourteen patients died shortly after the diagnosis [3].

The development of sarcoidosis or DISR after a malignancy diagnosis seems to have no impact on treatment or outcomes, and in 79–91% of patients, sarcoidosis resolves completely. SLR may even be considered a favorable prognostic factor, indicating a strong immune response and acting as a barrier to cancer cells [4,6,24].

However, when SLS is misdiagnosed, appropriate treatment is delayed, potential complications may arise, and the patient’s prognosis deteriorates [102].

## 10. Conclusions

In conclusion, the complex relationship between sarcoidosis and lymphoma, known as the sarcoidosis-lymphoma syndrome, reveals a multi-faceted interplay of genetic, environmental, and immunological factors. Distinguishing sarcoidosis from lymphoma is challenging due to overlapping clinical and imaging features. While advanced imaging techniques like FDG-PET/CT and MRI offer valuable insights, histological confirmation remains crucial. The co-existence of both conditions complicates diagnosis and management, requiring a multi-disciplinary approach to ensure accurate diagnosis and effective treatment. Evidence suggests that chronic inflammation, immune dysregulation, and specific genetic predispositions, including shared HLA alleles and microRNA patterns, may contribute to this association. Additionally, environmental exposures and infectious agents, while not conclusively linked to both conditions, appear to influence disease susceptibility. Immunosuppressive therapies, essential for managing sarcoidosis, introduce potential risks of malignancy, particularly in individuals with prolonged exposure. Furthermore, sarcoid-like reactions (SLRs) highlight the possibility of paraneoplastic manifestations and stress the importance of distinguishing confirmed sarcoidosis from granulomatous responses associated with malignancy. A deeper understanding of the shared molecular pathways, such as the JAK/STAT and oxidative stress mechanisms, may not only elucidate the development of sarcoidosis-lymphoma syndrome but also improve therapeutic approaches, including targeted immunomodulatory treatments. This highlights the necessity for vigilant monitoring and personalized management in patients with sarcoidosis, especially those requiring long-term immunosuppression, to balance therapeutic benefits with cancer risk.

## 11. Key Points

- Sarcoidosis-lymphoma syndrome describes the link between sarcoidosis and lymphoproliferative diseases, often seen in middle-aged individuals, with Hodgkin’s disease being the most common lymphoma type;
- Differentiating sarcoidosis from lymphoma is challenging due to overlapping features, requiring a combination of advanced imaging techniques, histological verification, and careful clinical assessment;
- Sarcoidosis may develop before, after, or alongside cancer. Key contributing factors include genetic predisposition, immunosuppressive treatment, and sarcoid-like reactions (SLRs) triggered by tumors;
- Granulomas form in sarcoidosis due to a cell-mediated immune response, which can also occur around tumor antigens, showing immunological overlap with lymphoma;
- Chronic inflammation and immune dysregulation drive both sarcoidosis and cancer risk, with macrophage polarization (M1/ M2 types) and cytokine elevations playing significant roles;
- SLRs are granulomas in cancer patients without systemic sarcoidosis, often triggered by cancer therapies or by cancer cells and their microenvironment, and may indicate an immune response to cancer;
- Shared genetic markers like microRNAs and HLA alleles link sarcoidosis and lymphoma, suggesting common immunological pathways;
- Risk factors like smoking, obesity, and certain infections (e.g., EBV, HIV for lymphoma, bacteria for sarcoidosis) influence disease development in each condition;
- Long-term use of corticosteroids and other immunosuppressants in sarcoidosis may increase lymphoma risk, necessitating close monitoring in treated patients. These points summarize the complex interactions between sarcoidosis, lymphoma, immune responses, genetic predispositions, and environmental influences.

## Figures and Tables

**Table 1:** This table lists the different risk factors of NHL, HL, and sarcoidosis. The specific type of malignancy they predispose to is indicated in parentheses for certain risk factors associated with lymphoma. Abbreviations used: NHL: non-Hodgkin lymphoma, HL: Hodgkin lymphoma, FL: follicular lymphoma, CLL: chronic lymphocytic leukemia, TCE: trichloroethylene, UV: ultraviolet, BMI: body mass index. According to references: [69,70,71,72,73].

NHL	HL	Sarcoidosis
Smoking (FL)	Smoking	Decreased sun exposure
Hair Dye ^1^ (FL, CLL)	Eczema	Inhalation of organic bioaerosols (musty odors, industrial organic dusts)
UV Radiation	Ionizing Radiation (especially Uranium)	Inhalation of inorganic aerosol exposures (several metal dusts) ^3^
Dietary Fat		Wood stove, fireplace use
Dessert Foods		Exposure to photocopier toner
rCarbohydrates (B-cell lymphoma)		Silica exposure
Broiled meat		Man-made mineral fiber exposure
Solvents (especially benzene and TCE)		Silicate exposure
Some pesticides		Working with vegetable dust
Higher BMI	Higher BMI	Higher BMI
Farmers ^2^		Living/Working on a Farm ^2^
Blood Transfusion (nodal B-CLL, high-grade extranodal lymphomas)		Working with high humidity

^1^ Frequent use before 1980 for over 25 years and especially permanent, dark dye. ^2^ Concerning occupation, farmers and agricultural workers have the most substantial evidence for elevated NHL risk, suggesting that chemical exposure or viral agents are to blame. ^3^ beryllium, zirconium, titanium, nickel, chromium, cobalt, silicon, earth elements, and aluminum.

**Table 2:** This table lists the different protective factors of NHL, HL, and sarcoidosis. The specific type of malignancy they are known to prevent is noted in parentheses for certain lymphoma-related factors. Abbreviations used: DLBCL: Diffuse large B-cell lymphoma, FL: follicular lymphoma, EBV: Epstein-Barr virus, BMI: body mass index. According to references: [71,72,73,74].

NHL	HL	Sarcoidosis
UV Radiation (B-cell subtypes, DLBCL)	UV Radiation (EBV-positive HL)	Smoking
Vitamin C (FL)	Physical activity (younger women)	
Dietary B12	Higher BMI (Older Women)	
Vitamin B6, Methionine, Folate	Low-dose aspirin use ^1^	
Other Antioxidants: Dietary Manganese, Proanthocyanidins, Alpha-Carotene		
High intake of some vegetables (cruciferous vegetables) and fruits		
Sun Exposure		
Atopic Diseases		
Blood Transfusion		
Alcohol		

^1^ Protective factor only for never/rare users of non-aspirin non-steroidal anti-inflammatory drugs (NSAIDs).
